# S-Pred: protein structural property prediction using MSA transformer

**DOI:** 10.1038/s41598-022-18205-9

**Published:** 2022-08-16

**Authors:** Yiyu Hong, Jinung Song, Junsu Ko, Juyong Lee, Woong-Hee Shin

**Affiliations:** 1 Arontier Co., Seoul, 06735 Republic of Korea; 2 Division of Chemistry and Biochemistry, Department of Chemistry, Kangwon National University, Chuncheon, 24341 Republic of Korea; 3 Department of Chemistry Education, Sunchon National University, Suncheon, 57922 Republic of Korea; 4 Department of Advanced Components and Materials Engineering, Sunchon National University, Suncheon, 57922 Republic of Korea

**Keywords:** Structural biology, Molecular modelling, Machine learning, Protein structure predictions

## Abstract

Predicting the local structural features of a protein from its amino acid sequence helps its function prediction to be revealed and assists in three-dimensional structural modeling. As the sequence-structure gap increases, prediction methods have been developed to bridge this gap. Additionally, as the size of the structural database and computing power increase, the performance of these methods have also significantly improved. Herein, we present a powerful new tool called S-Pred, which can predict eight-state secondary structures (SS8), accessible surface areas (ASAs), and intrinsically disordered regions (IDRs) from a given sequence. For feature prediction, S-Pred uses multiple sequence alignment (MSA) of a query sequence as an input. The MSA input is converted to features by the MSA Transformer, which is a protein language model that uses an attention mechanism. A long short-term memory (LSTM) was employed to produce the final prediction. The performance of S-Pred was evaluated on several test sets, and the program consistently provided accurate predictions. The accuracy of the SS8 prediction was approximately 76%, and the Pearson’s correlation between the experimental and predicted ASAs was 0.84. Additionally, an IDR could be accurately predicted with an F1-score of 0.514. The program is freely available at https://github.com/arontier/S_Pred_Paper and https://ad3.io as a code and a web server.

## Introduction

Proteins play an important role in biological processes, and their structures are closely linked to their functions. To characterize their structures, various experimental methods, such as X-ray crystallography, nuclear magnetic resonance spectroscopy, and cryogenic electron microscopy have been employed. However, because experimental protein conformation is difficult to obtain, the gap between the number of experimentally solved protein structures and the number of determined amino acid sequences is gradually increasing^1^. As of February 2022, approximately 225 million sequences have been compiled in the UniProt database^2^, and the structures of 108 thousand unique proteins structures have been deposited in the Protein Data Bank (PDB)^3^. Several protein structure prediction algorithms have been developed and are being routinely utilized to bridge the sequence-structure gap.

Several methods exist for extracting protein structural features from the amino acid sequence, known as the primary structure of proteins, to study its function. In 1961, Anfinsen^4^ discovered that a protein's tertiary structure is encoded by its amino acid sequence. Based on this observation, numerous approaches for predicting the structural properties of proteins, such as secondary structures, accessible surface areas (ASAs), and intrinsically disordered regions (IDRs), have been developed^5–17^. These features can also be useful for protein structural modeling by providing insights into local structures.

Since the early 2010s, numerous structural feature prediction approaches have been proposed, and as structural datasets expand, machine learning techniques, especially deep learning, have become more powerful. SPOT-1D^8^ uses long short-term memory (LSTM) and ResNet hybrid models to predict the eight-state secondary structures (SS8), ASAs, backbone dihedral angles, and contact numbers. The program uses a position-specific scoring matrix (PSSM) from multiple sequence alignment (MSA) and the predicted contact map from SPOT-Contact as input features. SPOT-Disorder^9,10^ predicts IDRs by employing multiple models sequentially, such as IncReSeNet, LSTM, and fully linked topological segments. The software uses both PSSM and structural information predicted by SPOT-1D to predict the disordered regions. NetSurfP-2.0^14^ uses a convolutional neural and LSTM from the protein sequence profile to predict the secondary structures, relative surface areas, IDRs, and backbone dihedral angles. MUFOLD-SS uses inception-inside-inception networks to predict the secondary structure from PSSM and seven physicochemical attributes of amino acids^16^. AUCpreD^17^ predicts IDRs using a convolutional neural network, considering seven physicochemical properties of amino acids, predicted secondary structures, solvent-accessible areas, and PSSM as input features.

In this paper, we present a new structural feature prediction method, S-Pred, which uses an LSTM and MSA Transformer^18^ for feature extraction from the MSA. The MSA Transformer is an unsupervised protein sequence language model introduced by Rao et al.^18^, which uses the MSA of a query sequence instead of a single amino acid sequence. The key attribute of this model is the use of row and column attentions for a given MSA and masked language model objectives. This model was successful in predicting long-range contacts between residues. Ultimately, this demonstrates that this protein language model is effective in extracting protein properties from MSA profiles. S-Pred uses the extracted features from the MSA Transformer and an LSTM to predict three structural features: SS8s, ASAs, and IDRs. The results indicate that S-Pred successfully predicts structural features accurately, and its performance is comparable to or superior to that of other state-of-the-art programs.

## Methods

### Network architecture

**Figure 1:**
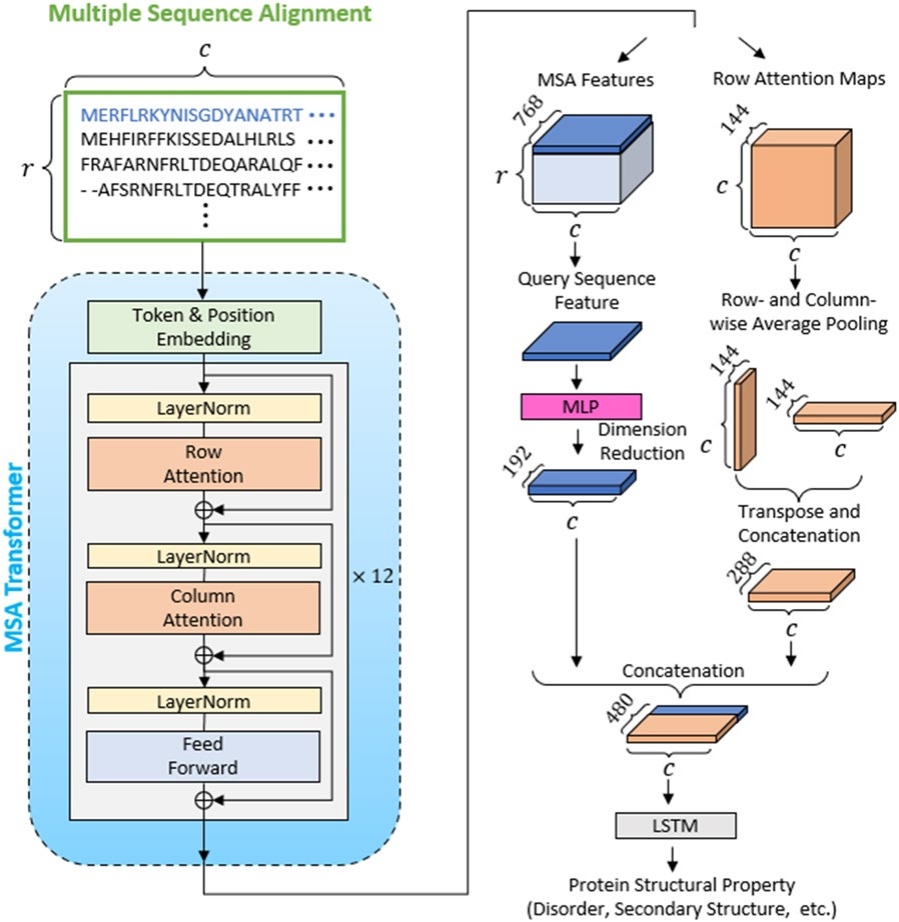
Architecture of the S-Pred method. The MSA Transformer extracts features and row attention maps from the input MSA of a query sequence. Next, through a series of transformations, the MSA features corresponding to the query sequence and the row attention maps are combined to 1D feature vectors. The 1D feature vectors are then input in an LSTM to predict protein structural properties including, SS8, ASA, and IDR.

The overall architecture of the algorithm is illustrated in Fig. 1. The input to the network is the MSA of a query sequence. The MSA is defined as an $\mathrm{r}\times \mathrm{c}$ matrix, where $\mathrm{r}$ is the number of sequences, and $\mathrm{c}$ is the sequence length. Through the token and position embedding of the MSA Transformer, the matrix is embedded into an $\mathrm{r}\times \mathrm{c}\times 768$ tensor, which is the input and output of each attention block^18^. The MSA Transformer is composed of a stack of 12 attention blocks. The attention blocks consist of three layers: two attention layers (row and column attention layer) with 12 attention heads and one feed-forward layer. Herein, for each layer, a normalization operation was performed.

A one-dimensional (1D) feature vector for each residue of a given sequence was generated by extracting two feature types from the MSA Transformer. The first was labelled as MSA features, which is the output tensor of the last attention block with dimensions of $\mathrm{r}\times \mathrm{c}\times 768$. From the MSA features, only the row that corresponded to the query sequence was selected, yielding a $1\times \mathrm{c}\times 768$ dimensional tensor. The dimension of the tensor was further reduced to $1\times \mathrm{c}\times 192$ using a multilayer perceptron (MLP) neural network consisting of three linear layers with 768, 384, and 192 neurons.

The second feature was the row attention map from every attention head. The MSA Transformer is composed of attention layers derived from 12 blocks with 12 attention heads. Thus, 144 attention maps were collated in the shape of a $\mathrm{c}\times \mathrm{c}\times 144$ tensor. The average pooling operation was applied to the row- and column-wise tensor to obtain $1\times \mathrm{c}\times 144$ and $\mathrm{c}\times 1\times 144$ tensors. The second tensor was transposed and concatenated with the first to yield a $1\times \mathrm{c}\times 288$ tensor.

The aforementioned two features were further concatenated to produce a $1\times \mathrm{c}\times 480$ tensor. Consequently, each residue for a given query sequence had a 480-dimensional feature vector. The feature tensors were sequentially proceeded to a set of two LSTM layers with 256 hidden units and a classification layer designed to predict the structural properties of each residue of a protein.

Three independent models were trained for the three structural properties by changing only the output neuron sizes of the final classification layer. Here, the classification layer possessed eight output neurons for SS8 and a single output neuron for ASA and IDR prediction.

### MSA generation

A procedure similar to that used for the MSA Transformer was used to generate the MSA of a query protein^18^. HHblits 3.3.0^19^ with the uniclus-ter30_2017_10^20^ and BFD^21^ databases were used to generate the MSA of the query sequence. The maximum number of sequences used in the MSA was set to 256. If the number of homologous sequences detected by HHblits was greater than the maximum number, 256 sequences were selected by minimizing the diversity.

### Training and inference

The parameters of the MSA Transformer have been fixed and described by Rao et al.^18^. The parameters of the other networks (i.e., MLP, LSTM, and classification layer) were trained using a batch size of 16 with gradient accumulation steps and a learning rate of 1e-3 using the RAdam optimizer^22^. Three independent models were used to individually train the SS8, ASA, and IDR datasets. The SS8, ASA, and IDR datasets were classified as multi-classification, regression, and binary classification, respectively. Thus, three different loss functions were generated including categorical cross-entropy for the SS8 data, mean squared error for the ASA data, and binary cross-entropy for the IDR data. For the ASA dataset, the values were divided by 200 prior to training to make the values smaller. All the models were trained for approximately 15 epochs using an NVIDIA Quadro RTX 8000 graphics processing unit (GPU) (48 GB).

An MSA subsampling strategy was applied during training. This was done not only for data augmentation to train a robust model, but also to prevent the GPU from running out of memory when filled with a large MSA. MSA rows were randomly selected for subsampling, with a maximum of ${2}^{14}/c$ and a minimum of 16, to ensure that the query sequences in the first row were always included. Large proteins with a length greater than 1023 residues were discarded during training. The MSA was subsampled with 256 sequences at the inference stage by adding the sequence with the lowest average Hamming distance.

### Datasets for SS8 and ASA

Datasets from Hanson et al.^8^ were used to train and test the SS8 and ASA networks. From the PIECES server^23^, 12,450 proteins with a resolution < 2.5 Å, R-free < 1, and sequence identity cutoff of 25% were extracted in February 2017. The proteins were further classified into three datasets: training (10,200 proteins), validation (1000 proteins), and test (1250 proteins) datasets. The authors generated another test set composed of 250 proteins, which were deposited in the PDB^3^ between 1/1/2018 and 7/16/2018 under identical conditions of resolution, R-free, and sequence identity. The two test sets were labeled as TEST2016 (collected February 2017) and TEST2018.

In addition, S-Pred’s SS8 prediction module was further tested on the Critical Assessment of protein Structure Prediction 13 (CASP13) dataset. To compare with other programs, the target list was kept the same as the DNSS2 paper^24^. Since the CASP13 was held in 2018 and our training set was culled in Feb. 2017, there is no overlap between the two datasets. The proteins were categorized as template-based modeling (TBM) and free modeling (FM) following the official classification.

### Datasets for IDR

Datasets from the SPOT-Disorder study were used to obtain the IDR prediction model^9^. Zhou et al. collected 4229 proteins from DisProt 5.0^25^, composed of 4157 X-ray crystallography structures and 72 fully disordered proteins. These data were divided into 2700 proteins for training, 300 proteins for validation, and 1229 proteins for testing. Proteins that contained more than 1023 amino acids were eliminated because the MSA Transformer could not treat large proteins. Thus, the remaining 2689 proteins were used for training, 300 proteins were used for validation, and 1225 proteins were used for testing. To compare with methods other than SPOT-Disorder, the IDR prediction model was also tested on three independent datasets: SL250, DisProt228^10^, and the Critical Assessment of Protein Intrinsic Disorder (CAID) dataset^26^. As its name suggests, SL250 is composed of 250 proteins and re-annotated DisProt proteins that include reliable disordered and ordered regions. DisProt228 contains 228 proteins collated from DisProt 7.0 but not included in DisProt 5.0; therefore, the proteins were not included in any training, validation, or test sets. The last dataset used was the CAID^26^. CAID is a blind IDR prediction experiment organized by Dr. Tosatto of Padua University. The dataset was constructed using 646 proteins that were annotated in the DisProt database from June 2018 to November 2018 and have been evaluated using 32 IDR prediction programs. The complete CAID prediction data were collected, and only the sequences predicted by all 33 predictors (32 from CAID and S-Pred) were retained (550 proteins) to provide a reasonable performance comparison.

### AlphaFold2 dataset

The aim of S-Pred is to predict the structural features of a protein from its sequence to study the molecule’s structure and function. In the recent CASP, AlphaFold2^27^ (AF2) showed the highest performance, which was around twice as high as the second-placed group^28^. Additionally, the AF2 predicted model is used to resolve the phasing problem in many proteins. Sequence-based structural feature prediction techniques might become obsolete due to AF2's strength. We gathered a dataset called the AF2 dataset to see if S-Pred might provide any further value beyond AF2 prediction. 2176 protein structures deposited in PDB database^3^ from 4/26^/^2022 to 6/28/2022 were collected. To remove the redundancy, PIECES server^23^ were used with the conditions of sequence identity < 25%, resolution < 2.5 Å, R value < 0.25, and sequence length between 50 and 1000, resulting 263 chains. We searched AlphaFold Protein Structure Database^29^ (Accessed 7/12/2022) with the UniProt ID^2^ of the chains and downloaded 92 structures. The structural features of predicted structures and corresponding crystal structures were calculated using DSSP^30^ and compared with S-Pred prediction results from their sequences. The qualities of models were measured by using TM-align^31^.

### Evaluation metrics and performance comparison

Because the S-Pred method predicts three different features (i.e., SS8, ASA, and IDR), several metrics and methods were utilized to evaluate its performance. For SS8, accuracy was evaluated to compare overall performance against previous data obtained from Hanson et al.^8^, Uddin et al.^11^, and Guo et al*.*^24^. To further investigate the performance of S-Pred on each secondary structure state, the precision, recall, and F1-score were calculated for the TEST2016 dataset. Pearson’s correlation coefficient (PCC) was used to assess the performance of the ASA model and was compared with that obtained in the study by Hanson et al.^8^. The IDR model was evaluated by calculating the area under the receiver operating curve (AUC_ROC_), Matthew’s correlation coefficient (MCC), and F1-score. The performance of the S-Pred IDR prediction model was compared with that of several methods presented in the SPOT-Disorder2^10^ and CAID studies^26^.

## Results and discussion

### SS8 prediction

The secondary structure of a protein is defined by the local structure of its peptide backbone. In general, the local backbone conformation is categorized into three states (SS3): helix (H), strand (E), and coil (C). Kabsch and Sander^30^ introduced a more detailed SS8 classification: α-helix (H), 3_10_-helix (G), π-helix (I), β-strand (E), isolated β-bridge (B), turn (T), bend (S), and others (C). H, G, and I in the SS8 classification correspond to the helix states in SS3, E and B are members of the strand states of SS3, and the remaining (T, S, C) are classified as the coil states of SS3. As the secondary structure provides information on the local conformation, SS8 may provide information for structure prediction that is more useful than the information provided by SS3 when used as a classifier.

The accuracy of the S-Pred method in classifying the validation dataset was 0.780, which is comparable with that of the state-of-the-art SS8 prediction methods, SPOT-1D (0.776)^8^ and SAINT (0.782)^11^, using the same training, test, and validation datasets. The SPOT-1D and SAINT programs use identical input features: 50 features of a PSSM derived from PSI-BLAST^32^ and HHblits^19^, seven physicochemical properties such as Van der Waal’s volume and polarizability, and a contact prediction map from SPOT-Contact^33^. SPOT-1D operates by employing an ensemble of LSTM networks in a bidirectional recurrent neural network and ResNet hybrid models^8^, whereas SAINT utilizes an ensemble of a self-attention mechanism with Deep3I network^11^. In contrast, S-Pred only requires an MSA constructed from HHblits and uses a single model to predict the SS8s.

**Figure 2:**
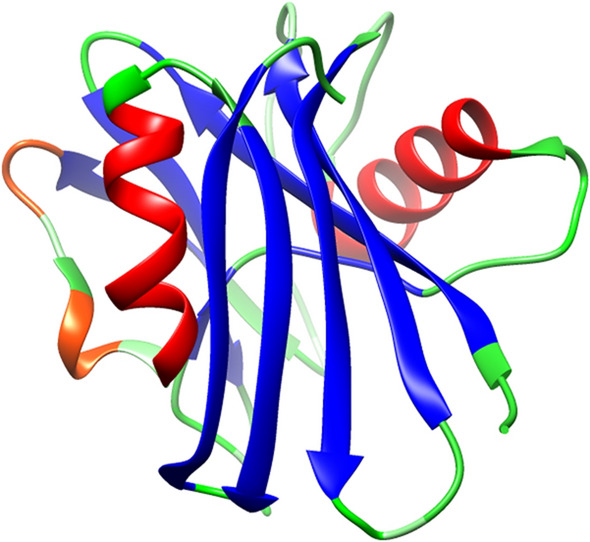
S-Pred SS8 predictions mapped on the 7,8-dihydro-8-oxoguanine triphosphatase (PDB ID: 5WS7) structure. The color codes for α-helix (H), 3_10_-helix (G), β-strand (E), turn (T), bend (S), and others (C) are red, orange-red, blue, green, light green, and lime green, respectively. It should be noted that none of the residues were predicted as π-helices (I) or β-bridges (B).

The performance of S-Pred on the TEST2016 and TEST2018 datasets for SS8 prediction is presented in Table 1. S-Pred demonstrates a prediction accuracy of 0.776 for the TEST2016 set, which ranks 2nd among the tested methods in SPOT-1D^8^ and SAINT^11^ papers. The prediction accuracy of S-Pred is similar to that of SAINT, which is the best-performing method, and outperforms the SPOT-1D method. Additionally, S-Pred outperforms SPOT-1D-base, which utilizes an ensemble collection of nine models trained without contact map prediction, and SAINT-base, which uses a single model. With the TEST2018 set, the S-Pred method achieves the highest accuracy (0.764), whereas the accuracy of SAINT is slightly lower (0.761). Interestingly, S-Pred is the best-performing program in terms of the accuracy for SS3 prediction (0.865, Supporting Information Table S1). An example of SS8 prediction using the 7,8-dihydro-8-oxoguanine triphosphatase sequence (PDB ID: 5WS7) from the TEST2016 dataset is illustrated in Fig. 2.

**Table 1:** Comparison of the SS8 accuracy obtained from several methods on the TEST2016 and TEST2018 datasets.

Method	TEST2016	TEST2018
S-Pred	0.776	**0.764**
SPIDER-3-Single^a^	N/A	0.598
DNSS2	N/A	0.655
RaptorX^a^	N/A	0.704
POTTER-5^a^	N/A	0.732
MUFOLD-SS^b^	0.756	0.737
NetSurfP-2.0^b^	0.757	0.730
SPOT-1D-base^a^	0.760	0.743
SPOT-1 Da	0.771	0.754
SAINT-base^b^	0.762	0.745
SAINT^b^	**0.777**	0.761

The data acquired from other methods except DNSS2 were obtained from Hanson et al*.*^8^ and Uddin et al*.*^11^. The method that performs the best is represented in bold.

^a^Data adapted from Hanson et al.^8^.

^b^Data adapted from Uddin et al.^11^.

To provide a better understanding, we further investigated the performance of the individual secondary structure state from the TEST2016 dataset with regards to precision, recall, and F1-score, and the results are presented in Table 2. The F1-score is a harmonic average of the precision and recall, balancing the two metrics, thus widely used for imbalanced data. As can be observed, the S-Pred method performs better than the other methods in predicting four of the eight secondary structure classes (H, E, G, and C). Interestingly, our methodology produces an F1-score higher than 0.6 for states with more than 30,000 residues (H, E, T, and C) in the dataset. For non-ordinary states, such as B, G, I, and S, the program generates F1-scores lower than 0.5. SAINT and SPOT-1D, which use two-dimensional contact map information as the primary additional input features, perform better than S-Pred for non-ordinary secondary structure states. Because the secondary structure is defined by the local hydrogen bond patterns of the backbone, two-dimensional contact map information may be useful in predicting non-ordinary secondary structures. This predictive trend on non-ordinary states was also observed in studies conducted by Wang et al.^12^ and Zhang et al.^13^ that used deep learning for SS8 prediction. This result suggests that S-Pred may improve its performance when contact information is included.

**Table 2:** Precision, recall, and F1-score for individual secondary structure states obtained from the TEST2016 dataset.

Label	Precision					Recall					F1-score				
	SP^a^	SA^b^	S1^c^	NS^d^	MU^e^	SP^a^	SA^b^	S1^c^	NS^d^	MU^e^	SP^a^	SA^b^	S1^c^	NS^d^	MU^e^
H (98139)	**0.886**	0.879	0.884	0.885	0.868	**0.953**	0.948	0.941	0.933	0.943	**0.918**	0.912	0.911	0.908	0.904
B (3018)	0.660	**0.760**	0.671	0.650	0.609	0.101	0.104	0.097	0.070	**0.115**	0.176	**0.183**	0.169	0.126	0.193
E (62657)	**0.859**	0.843	0.852	0.822	0.850	0.874	0.887	0.878	**0.903**	0.842	**0.866**	0.864	0.865	0.861	0.846
G (10770)	**0.588**	0.581	0.547	0.536	0.519	**0.394**	0.390	0.375	0.334	0.348	**0.471**	0.467	0.445	0.412	0.417
I (47)	0.235	**1.000**	**1.000**	0.044	0.857	0.085	**0.447**	0.128	0.426	0.383	0.125	**0.618**	0.227	0.079	0.529
T (32297)	0.622	**0.663**	0.641	0.615	0.631	**0.648**	0.618	0.612	0.585	0.586	0.635	**0.639**	0.626	0.599	0.608
S (23466)	**0.674**	0.639	0.624	0.579	0.589	0.286	**0.367**	0.337	0.278	0.313	0.402	**0.466**	0.438	0.376	0.409
C (57483)	0.640	**0.648**	0.631	0.613	0.607	**0.748**	0.731	0.741	0.704	0.727	**0.690**	0.687	0.682	0.655	0.662

The numbers in parentheses represent frequencies of the secondary structure states. The data from all other methods except S-Pred were obtained from Uddin et al.^11^. The method that performs the best is represented in bold.

^a^S-Pred (SP).

^b^SAINT (SA).

^c^SPOT-1D (S1).

^d^NetSurfP-2.0 (NS).

^e^MUFOLD-SS (MU).

S-Pred also showed a good performance on the CASP13 benchmark (Table 3). Among the tested methods, S-Pred showed the second highest accuracy in the All and TBM category (0.724 and 0.738, respectively), comparable to DNSS2, the top-performed method (0.727 and 0.753 for the All and TBM category). Interestingly, our program performed best in the FM category (0.714), which is composed of proteins with few available templates to model the structure. The difference in accuracy between the TBM and FM categories is only 0.024, the smallest gap among the tested methods. This implies that S-Pred could perform consistently although there are few or no structural templates.

**Table 3:** Prediction of SS8 on CASP13 dataset. All values except S-Pred were taken from Guo et al.^24^.

Method	All	TBM	FM
S-Pred	0.724	0.738	**0.714**
SSPro5.2	0.644	0.664	0.640
DeepCNF	0.665	0.689	0.653
MUFOLD	0.667	0.684	0.661
Porter 5	0.677	0.709	0.657
DNSS2	**0.727**	**0.753**	0.710

The method that performs the best is represented in bold.

### ASA prediction

A key structural feature of a protein residue is its ASA. This metric is regarded as a significant factor in protein folding and stability^34^. The ASA metric can be used to classify the residue as buried inside a protein or exposed on the surface. Thus, for protein structure prediction, the ASA metric is crucial in indicating the location of the residue. The S-Pred predictive performance was evaluated by the PCC for the predicted and real ASA values, which were calculated using the DSSP algorithm^30^. The PCC for the validation set was 0.850, which was higher than that of SPOT-1D (0.823) with the same dataset.

**Figure 3:**
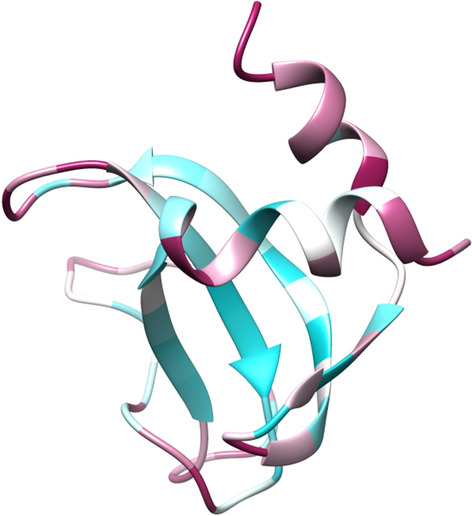
S-Pred ASA predictions mapped on the structure of nuclear fusion protein BIK1 (PDB ID: 6FC6). The residues are represented using a gradient color scale from cyan (buried) to maroon (exposed).

Table 4 compares the performance on the TEST2016 and TEST2018 datasets. As can be observed, PCC values of S-Pred for the TEST2016 (0.843) and TEST2018 (0.831) datasets are larger in magnitude than the PCCs obtained from the other computational methods on the same datasets. Similar to the validation set, S-Pred produces larger PCCs than SPOT-1D. However, unlike SPOT-1D, which requires PSSM as well as physicochemical features and contact map information, S-Pred only requires an MSA from HHblits and provides improved performance. Zhou et al. compared the performance of the retrained SPIDER3 and SPOT-1D algorithms by calculating PCCs on the same training set^8^ because the size of the SPOT-1D training set is twice as large as that of SPIDER3 (4590 proteins). The PCC obtained from SPIDER3 increased to 0.796, from 0.76, but was still lower than that obtained from S-Pred. NetSurfP-2.0, which uses an MSA from HHblits^14^ similar to S-Pred, also generates lower PCCs than our computational method. An example of ASAs predicted by S-Pred is displayed on chain A of PDB ID 6FC6, the nuclear fusion protein BIK1, from TEST2018 (Fig. 3).

**Table 4:** Prediction of ASAs and comparison among methods.

Method	TEST2016	TEST2018
S-Pred	**0.843**	**0.831**
SPIDER-3	0.787	0.768
NetSurfP-2.0	N/A	0.801
SPOT-1D-base	0.813	0.799
SPOT-1D	0.816	0.803

PCCs between the predicted and experimental values. The PCCs obtained from methods except S-Pred were used from the study conducted by Hanson et al.^8^. The method that performs the best is represented in bold.

SPOT-1D is an improvement on the SPOT-1D-base method because it incorporates the contact map information from SPOT-Contact. As the contact information contains the number of residues surrounding the target amino acid, it may provide additional information on ASAs. This implies that the performance of S-Pred in predicting ASAs can potentially be improved if the contact information is used as a supplementary input feature.

### IDR prediction

IDRs and intrinsically disordered proteins (IDPs) do not possess fixed three-dimensional structures. IDPs and IDRs are involved in various biological processes because they can adopt multiple conformations and bind to several protein partners. According to a recent study^35^, eukaryotic proteomes are more disordered than other domains, with a 20.5% disordered content. In addition, IDRs are linked to various human diseases, such as cancers and Alzheimer’s disease; therefore, they have been employed as potential drug targets. From a structural prediction perspective, eliminating the IDRs before modeling can be helpful in excluding regions that cannot be successfully modeled. Thus, IDR prediction is crucial for both the biological function prediction and computational modeling of proteins.

**Figure 4:**
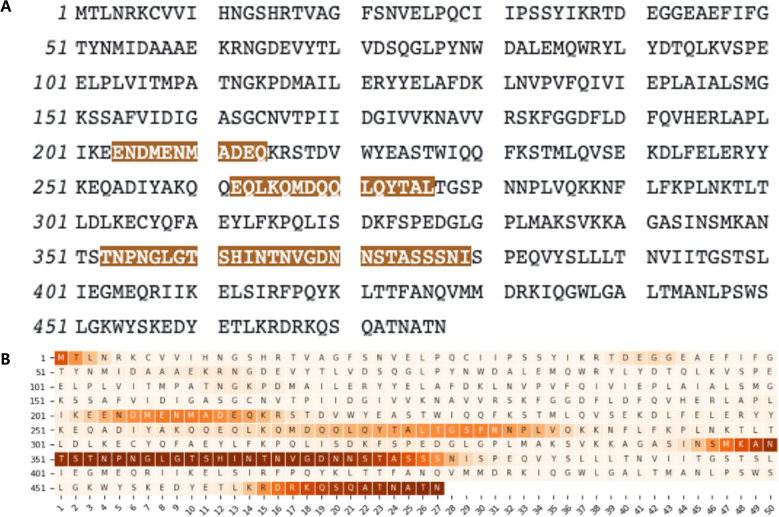
S-Pred IDR prediction for the DisProt ID DP00874 protein. (**A**) DisProt annotations. Disordered annotated residues are highlighted in brown. (**B**) S-Pred prediction. The probability of disorder is represented as a function of the background color intensity. Thus, the higher probability of disorder is portrayed as a darker brown color.

The S-Pred model produced AUC_ROC_ values of 0.929 and 0.914 for the validation and test sets, respectively. For comparison, two additional independent datasets (i.e., SL250 and DisProt228) were employed (Table 5). The performance of S-Pred on these datasets was evaluated using two metrics, AUC_ROC_ and MCC. S-Pred and the other state-of-the-art methods performed comparably on both test sets. For the SL250 set, S-Pred ranked 2nd for both the AUC_ROC_ and MCC metrics (0.884 and 0.650, respectively), whereas for the DisProt228 dataset, it ranked 2nd for AUC_ROC_ (0.797) and 4th for MCC metrics (0.457). An example of IDR prediction using the DisProt ID DP00874 (actin-related protein 7) is illustrated in Fig. 4. As can be observed, S-Pred predicts three disordered regions that are in a location similar to that of the annotated regions.

**Table 5:** Comparison of IDR predictions by several methods.

Set	Program	AUC_ROC_	MCC
SL250	S-Pred	0.884	0.650
	s2D	0.737	0.360
	MobiDB-lite	0.818	0.534
	DISOPRED2	0.825	0.508
	ESpritz-N	0.833	0.454
	ESpritz-D	0.843	0.555
	DISOPRED3	0.857	0.596
	ESpritz-X	0.859	0.566
	NetSurfP-2.0	0.869	0.572
	ACUpreD	0.869	0.605
	SPINE-D	0.875	0.599
	SPOT-Disorder	0.893	0.629
	SPOT-Disorder2	**0.901**	**0.679**
DisProt228	S-Pred	0.797	0.457
	s2D	0.727	0.267
	AUCpreD	0.748	0.434
	JRONN	0.753	0.379
	ESpritz-D	0.759	0.379
	MFDp2	0.768	0.371
	DISOPRED	0.771	0.406
	MobiDB-lite	0.772	0.422
	NetSurfP-2.0	0.774	0.421
	Espritz-N	0.776	0.432
	MFDp	0.776	0.357
	SPINE-D	0.786	0.423
	SPOT-Disorder	0.792	0.462
	ESpritz-X	0.796	0.476
	SPOT-Disorder2	**0.809**	**0.499**

The AUC_ROC_ and MCC metrics for methods other than S-Pred were obtained from the study conducted by Hanson et al.^10^. For the DisProt228 dataset, only sequence-profile-based methods are presented. The method that performs the best is represented in bold.

The top-performing method in both datasets was SPOT-Disorder2. SPOT-Disorder2 is a profile-based IDR prediction method that utilizes PSSM profiles from PSI-BLAST and HHblits^10^. In addition, it also employs 23 structural properties predicted by SPOT-1D (SS8, SS3, four sine and cosine values of backbone dihedral angles (θ, τ, φ, and Ψ), ASA, contact number, and two half-sphere exposure values) as input features^10^. The final prediction of SPOT-Disorder2 is based on a combination of five models. By contrast, S-Pred uses only an MSA as the input feature and a single model. Zhou et al. examined the effects of the structural input features predicted by SPOT-1D^10^. The AUC_ROC_ of Model 0 on the Mobi9414 test set was 0.943; however, it reduced to 0.920 when the features from SPOT-1D were omitted. This implies that the performance of S-Pred in IDR prediction can be potentially improved by incorporating structural features from the SS8 and ASA modules.

### CAID IDR prediction

The final benchmark was the CAID experiment. A probability threshold for estimating the IDR residue of each method was optimized to acquire the highest F1-score (*F*_max_), the same as in the original benchmark study^26^. After relabeling the residues with the thresholds, all metrics were examined. Table 6 presents the results obtained from the CAID experiment. As can be observed, among the 33 predictors, S-Pred ranks 2nd for all metrics: 0.514, 0.791, and 0.384 for the F1-score, AUC_ROC_, and MCC, respectively. The top-performing method is fIDPnn^15^, which is a meta-predictor of DFLpred, DisoRDPbind, and fMoRFpred using a neural network. Thus, S-Pred is the best performing method among non-meta-predictors.

**Table 6:** CAID benchmark results. Raw predictions were obtained from Necci et al.^26^.

Method	F1-score	AUC_ROC_	MCC
SPred	0.514	0.791	0.384
fIDPnn	**0.521**	**0.813**	**0.390**
SPOT-Disorder2	0.507	0.780	0.378
fIDPln	0.504	0.794	0.367
SPOT-Disorder	0.499	0.769	0.367
RawMSA	0.496	0.791	0.357
SPOT-Disorder-Single	0.488	0.769	0.348
AUCpreD	0.483	0.762	0.346
AUCpreD-np	0.481	0.761	0.335
ESpritz-D	0.479	0.775	0.332
MobiDB-lite	0.473	0.745	0.325
IUPred-long	0.473	0.752	0.324
IUPred2A-short	0.473	0.752	0.324
Predisorder	0.472	0.753	0.322
DisoMine	0.472	0.771	0.323
IsUnstruct	0.471	0.756	0.321
IUPred-short	0.471	0.751	0.321
IUPred2A-long	0.471	0.751	0.321
ESpritz-X	0.471	0.752	0.321
VSL2B	0.464	0.746	0.311
DISOPRED-3	0.463	0.727	0.313
JRONN	0.454	0.736	0.297
ESpritz-N	0.447	0.724	0.286
DynaMine	0.437	0.719	0.271
PyHCA	0.432	0.709	0.264
FoldUnfold	0.422	0.655	0.249
DisEMBL-465	0.413	0.695	0.239
S2D-1	0.401	0.668	0.216
S2D-2	0.387	0.653	0.192
DisEMBL-HL	0.375	0.657	0.174
DisPredict-2	0.368	0.634	0.158
GlobPlot	0.358	0.625	0.147
DFLpred	0.322	0.411	-0.029

The threshold value of each method for labeling IDR residues was optimized to obtain a *F*_max_. The method that performs the best is represented in bold.

The organizers of the CAID experiment also tested the predictors to determine whether they could predict fully disordered proteins, also referred to as IDPs. IDPs are targets of interest because they are difficult to be structurally characterized experimentally, but they possess unique biological functions. In the CAID benchmark set, proteins were considered as IDPs if the percentage of disordered annotated residues was higher than 95%. Using these criteria, 41 of 550 proteins were labeled as IDPs. Under the same conditions, the IDR prediction program predicted IDPs after labeling all amino acids as an input. The performance of the IDP prediction is presented in Supporting Information Table S2. As can be observed, S-Pred provides the most accurate IDP prediction (F1-score: 0.637; MCC: 0.609). Even if a more rigid IDP definition (99%) is used, the result does not substantially change. S-Pred is still the best IDP predictor with an F1-score and MCC of 0.652 and 0.624, respectively.

### Comparison with AF2 models

To investigate whether S-Pred could still provide valuable information beyond AF2, the most powerful tertiary structure prediction method, we collected 92 crystal structures from PDB that do not share high sequence identities (< 25%). S-Pred predicted SS8 and ASA from their UniProt sequences and compared them with AF2 models extracted from AlphaFold Protein Structure Database.

S-Pred reported that ASA PCC was 0.844 and SS8 accuracy was 0.778. In contrast, AF2 models outperformed S-Pred, scoring 0.900 and 0.915 for ASA PCC and SS8 accuracy, respectively. It is natural that AF2 has higher accuracy than S-Pred since AF2 might infer structural information from templates and contact maps, while S-Pred only has MSA as an input without structural information. When we examine the individual proteins, S-Pred performed better than AF2 in 7 (SS8) and 18 (ASA) cases out of 92 proteins. It's interesting to note that the AF2 models for the proteins that S-Pred surpassed are not very accurate. The seven proteins with greater S-Pred SS8 predictions have an average TM-score of 0.747, while the remaining proteins have an average TM-score of 0.959. The 18 proteins that S-Pred outperformed in ASA prediction have an average TM-score of 0.841, compared to 0.968 for the other proteins. In four protein cases, S-Pred performed better in both SS8 and ASA than AF2 models. The four proteins have a mean TM-score of 0.682, while the other proteins have an average TM-score of 0.955. This result implies that the quality of AF2 models might be improved by S-Pred prediction.

In addition, even though the AF2 model has improved accuracy, S-Pred is still valuable due of its quickness. S-Pred takes roughly 10 min to complete the input MSA construction for proteins with around 300 amino acids, and less than a second to complete the prediction. On the other hand, AF2 takes around 10 min for modeling and 4 h for MSA with a single GPU^36^. Protein structural characteristics might be quickly predicted and applied to studies like IDP prediction using S-Pred.

## Conclusions

Prediction of the structural properties of proteins from an amino acid sequence can aid in the prediction of the structure and biological function of proteins. In this paper, we report a novel structural feature prediction program called S-Pred. The program utilizes the MSA Transformer to obtain input features and predicts three structural features (SS8, ASA, and IDR) using an LSTM. This study demonstrated that our program successfully predicted all the three features, and the performance was better than or comparable with that of other state-of-the-art algorithms.

The benchmark result also provided useful information for improving the performance. For SS8 prediction, S-Pred failed to predict non-ordinary secondary structure states, such as isolated β-bridges and π-helices. In contrast, the SAINT and SPOT-1D methods successfully predicted these states because contact prediction was used as an input feature. For IDP prediction, SPOT-Disorder2, which demonstrated better performance in both the SL250 and DisProt228 benchmark sets, employed structural features predicted by SPOT-1D. Further studies should investigate approaches to improve the performance of S-Pred by incorporating components or modules from other prediction programs.

## Supplementary Information

Supplementary Information.

## Data Availability

The datasets analyzed during current study are available at https://doi.org/10.5281/zenodo.6873654.

## Code availability

The code can be accessed at https://github.com/arontier/S_Pred_Paper.
