# Supplementary material for: S-Pred: protein structural property prediction using MSA transformer
Source: Sci Rep. 2022 Aug 16;12:13891. doi: 10.1038/s41598-022-18205-9 (PMC9381718; doi:10.1038/s41598-022-18205-9)
Supplement: Supplementary file 1 — Supplementary Information. [file 41598_2022_18205_MOESM1_ESM.docx]

**Supporting Information for**

**S-Pred: Protein Structural Property Prediction Using MSA Transformer**

Yiyu Hong^1^, Jinung Song^1^, Junsu Ko^1^, Juyong Lee^1,2^ and Woong-Hee Shin^1,3,4*^

*^1^Arontier co., Seoul, 06735, Republic of Korea*

*^2^Department of Chemistry, Division of Chemistry and Biochemistry, Kangwon National University, Chuncheon 24341, Republic of Korea*

*^3^Department of Chemistry Education, Sunchon National University, Suncheon 57922, Republic of Korea*

*^4^Department of Advanced Components and Materials Engineering, Sunchon National University, Suncheon 57922, Republic of Korea*

**Table S1.** A comparison of TEST2018 SS3 accuracy. Data taken from Hanson *et al.* [8] except for S-Pred and DNSS2.

| Method | Accuracy |
| --- | --- |
| S-Pred | **0.865** |
| SPIDER-3-single | 0.726 |
| DNSS2 | 0.802 |
| RaptorX | 0.816 |
| PSRSM | 0.819 |
| SPIDER-3 | 0.838 |
| PORTER-5 | 0.841 |
| MUFOLD | 0.848 |
| NetSurfP-2.0 | 0.853 |
| SPOT-1D-base | 0.857 |
| SPOT-1D | 0.862 |

**Table S2.** Fully disordered protein prediction result with 95% cutoff and 99% cutoff.

|  | 95% Cutoff | | 99% Cutoff | |
| --- | --- | --- | --- | --- |
| Method | *F*1-score | MCC | *F*1-score | MCC |
| S-Pred | **0.637** | **0.609** | **0.652** | **0.624** |
| RawMSA | 0.605 | 0.572 | 0.625 | 0.596 |
| fIDPnn | 0.588 | 0.561 | 0.596 | 0.564 |
| SPOT-Disorder-Single | 0.559 | 0.523 | 0.562 | 0.525 |
| AUCpreD | 0.548 | 0.510 | 0.530 | 0.492 |
| SPOT-Disorder | 0.545 | 0.511 | 0.535 | 0.496 |
| fIDPln | 0.544 | 0.522 | 0.529 | 0.495 |
| MobiDB-lite | 0.534 | 0.543 | 0.491 | 0.528 |
| IUPred-long | 0.529 | 0.512 | 0.475 | 0.492 |
| SPOT-Disorder2 | 0.528 | 0.488 | 0.465 | 0.420 |
| VSL2B | 0.520 | 0.491 | 0.508 | 0.491 |
| IUPred2A-long | 0.484 | 0.485 | 0.456 | 0.486 |
| IUPred2A-short | 0.475 | 0.492 | 0.456 | 0.486 |
| AUCpreD-np | 0.462 | 0.421 | 0.468 | 0.429 |
| IUPred-short | 0.448 | 0.469 | 0.370 | 0.412 |
| DisoMine | 0.444 | 0.423 | 0.452 | 0.422 |
| IsUnstruct | 0.414 | 0.429 | 0.393 | 0.420 |
| DISOPRED-3 | 0.413 | 0.401 | 0.364 | 0.394 |
| DisPredict-2 | 0.400 | 0.355 | 0.447 | 0.401 |
| ESpritz-X | 0.400 | 0.358 | 0.382 | 0.352 |
| ESpritz-N | 0.400 | 0.438 | 0.415 | 0.479 |
| ESpritz-D | 0.376 | 0.374 | 0.379 | 0.373 |
| Predisorder | 0.370 | 0.412 | 0.314 | 0.376 |
| PyHCA | 0.370 | 0.412 | 0.217 | 0.338 |
| JRONN | 0.340 | 0.384 | 0.308 | 0.355 |
| FoldUnfold | 0.304 | 0.286 | 0.304 | 0.286 |
| DisEMBL-HL | 0.292 | 0.400 | 0.136 | 0.261 |
| DynaMine | 0.174 | 0.265 | 0.093 | 0.213 |
| DisEMBL-465 | 0.136 | 0.261 | 0.136 | 0.261 |
| DFLpred | 0.124 | -0.236 | 0.114 | -0.212 |
| S2D-1 | 0.083 | 0.091 | 0.089 | 0.139 |
| S2D-2 | 0.046 | 0.098 | 0.000 | -0.012 |
| GlobPlot | 0.000 | -0.012 | 0.000 | 0.000 |
